# Dysfunction and therapeutic potential of brown adipose tissue and batokines in obesity and type 2 diabetes mellitus

**DOI:** 10.3389/fendo.2026.1949288

**Published:** 2026-10-22

**Authors:** Hossein Mirmiranpour, Thomas M. Barber

**Affiliations:** 1 Endocrinology and Metabolism Research Center (EMRC), Vali-Asr Hospital, Tehran University of Medical Sciences, Tehran, Iran; 2 Division of Biomedical Sciences, Warwick Medical School, University of Warwick, Coventry, United Kingdom; 3 Warwickshire Institute for the Study of Diabetes, Endocrinology and Metabolism, University Hospitals Coventry and Warwickshire, Clifford Bridge Road, Coventry, United Kingdom; 4 Research Centre for Health and Life Sciences, Coventry University, Richard Crossman Building, Jordan Well, Coventry, United Kingdom

**Keywords:** batokines, brown adipose tissue, metabolism, obesity, type 2 diabetes mellitus

## Abstract

Obesity and Type 2 Diabetes Mellitus (T2D) remain key drivers of premature mortality, morbidity, and healthcare expenditure globally. Although we have witnessed dramatic recent advances in our therapeutic armamentarium for these conditions (most notably regarding the latest iterations of the Glucagon-like Peptide-1 [GLP-1] class of therapies), there remain important unmet needs in our current management approach. Brown adipose tissue (BAT) and its associated batokines (molecules release from BAT) plays a key role in the regulation of energy expenditure and glucose and lipid metabolism. Multiple lifestyle factors (most notably cold exposure and certain nutrients) can activate BAT via activation of the systemic nervous system (SNS). In the context of obesity and T2D, BAT activity is downregulated through multiple complex processes. This downregulation further promotes insulin resistance and dysregulation of metabolic regulation that contributes towards ongoing pathogenesis of these pernicious conditions, in a vicious circle. In such a scenario, it would be desirable to break this vicious circle through novel therapies that target the BAT-batokine axis. In recent times, there has been a transformation of our insights into this approach, particularly from data derived from rodent models. Beyond facilitating weight loss and improved metabolic efficiency and flexibility, such a novel approach to therapy that targets the BAT-batokine axis would improve glucose and lipid regulation but also have potential for organ-specific therapeutic benefits, including in the heart, lung, kidney, and liver. Furthermore, such an approach holds great promise to improve body composition (to reduce adiposity whilst preserving or even enhancing skeletal muscle mass and function), due to the therapeutic effects being independent of negative energy balance. The BAT-batokine axis has finally come of age.

## Introduction

Brown adipose tissue (BAT) is a specialized thermogenic organ that plays a critical role in whole body energy homeostasis (1, 2). BAT also regulates lipid and glucose utilization through the integration of complex neural, hormonal, and nutritional signals (3). Unlike white adipose tissue (WAT), the primary function of which is to store excess energy, BAT dissipates chemical energy as heat through ‘non-shivering thermogenesis’. This process is essential for maintaining body temperature (particularly in small rodents) and is an important contributor to overall energy expenditure (both basal and inducible) (4). BAT thermogenesis occurs through mitochondrial uncoupling, that in turn is mediated by ‘uncoupling protein 1’ (UCP1), a proton transporter located in the inner mitochondrial membrane (5). UCP1 dissipates the proton gradient generated by oxidative phosphorylation, releasing stored chemical energy as heat instead of utilising this to form adenosine triphosphate (ATP). This mechanism enables rapid substrate oxidation and increased energy expenditure (6, 7). The regulation of mitochondrial UCP1 expression and activity implicates transcriptional, post-translational, and lipid-mediated mechanisms (8, 9). Dysfunctional UCP1 signalling within BAT impairs its thermogenic capacity (10).

Beyond thermogenesis and metabolic regulation, BAT also functions as an endocrine organ that secretes bioactive molecules, collectively termed ‘batokines’. These factors include, among others, ‘fibroblast growth factor-21’ (FGF21), ‘interleukin-6’ (IL-6), ‘neuregulin-4’ (NRG4), ‘ependymid related protein 1’ (EPDR1), ‘phospholipid transfer protein’ (PLTP), ‘BAT-secreted peptide 1’ (BATSP1), ‘growth differentiation factor 15’ (GDF15), ‘12,13-dihydroxy-9Z-octadenoic acid’ (12,13 diHOME), and ‘C-X-C motif chemokine ligand 14’ (CXCL14) (11–13). Batokines mediate inter-organ communication and amplify the systemic metabolic benefits of activated BAT (14, 15). Batokines influence hepatic lipid metabolism, pancreatic insulin secretion, vascular function, and immune responses (15–17). Batokines such as NRG4 exert hepatoprotective effects, whilst others modulate renal fibrosis, pulmonary inflammation, and bile acid metabolism. These systemic benefits position activated BAT as a multi-organ protective tissue (18–20). Importantly, UCP1-dependent thermogenic activity modulates batokine secretion, while batokines in turn regulate mitochondrial biogenesis and UCP1 expression within BAT, forming a coordinated regulatory network (15, 16). This reciprocal interaction between activated BAT and metabolic regulation within the periphery (including the liver, adipose tissue and skeletal muscle) optimizes metabolic efficiency (21–23).

In humans, metabolically active BAT has been identified in adults and correlates inversely with adiposity and cardiometabolic risk. Furthermore, impaired BAT activity promotes metabolic inflexibility, weight gain and the development of obesity and obesity-related dysmetabolic conditions, including T2D (24, 25), typified by chronic energy surplus, insulin resistance, and low-grade inflammation (26). Clinical imaging studies reveal that obesity and T2D associate with reduced BAT mass and activity, including a diminishment of glucose uptake and thermogenic responsiveness in BAT (27). Conversely, activation of BAT (through cold exposure, β-adrenergic stimulation, or through exposure to dietary components such as chilli) improves insulin sensitivity, enhances glucose disposal, promotes lipid clearance, reduces vascular inflammation and improves cardiac energetics (28, 29). As such, BAT activation may lower cardiovascular risk. These responses can occur even in metabolically compromised states, highlighting the therapeutic plasticity of BAT. These observations provide proof of concept that restoring BAT function and thermogenesis may counteract the typical metabolic derangements observed in obesity and T2D (30, 31).

Negative or inconclusive clinical findings on BAT activation often stem from the fact that several preclinical BAT-activating mechanisms (such as robust thermogenic responses to β-adrenergic agonists) do not translate effectively to humans, where BAT volume is lower, responsiveness is weaker, and systemic metabolic effects are correspondingly modest. Preclinical studies consistently show strong thermogenic and metabolic improvements, but clinical trials frequently report minimal or no benefit, highlighting a gap between rodent BAT biology and human BAT physiology (32, 33).

Furthermore, BAT volume in adults is often small in volume and may possess limited thermogenic capacity. This may restrict the therapeutic potential of enhanced BAT-induced energy expenditure and would explain why stimulating BAT activity has shown limited effect on weight-loss in reported studies to date. Indeed, reported studies have demonstrated only a modest effect of BAT activation on energy expenditure and weight loss (34).

A challenge for the future will be to therapeutically manipulate BAT in a way that augments the volume of BAT (possibly through trans-differentiation of WAT to BAT, or through manipulation of mesenchymal stem cells that could differentiate into BAT), to provide a larger therapeutic target on which to focus BAT activators. This approach would likely improve the therapeutic potential of BAT activation through a greater impact on energy expenditure and reduction of adiposity.

The activation of BAT and the associated release of batokines (either through pharmacological or through lifestyle strategies) are novel and promising therapeutic targets for obesity and related dysmetabolic conditions such as T2D (18, 19, 35–37). These key insights demand our attention. In this concise review, we provide an overview of BAT physiology including the role of batokines in metabolic regulation. We then discuss the role of BAT dysfunction in the pathogenesis of obesity and T2D. Finally, we discuss the therapeutic potential of BAT activation within vital organs.

## Overview of BAT physiology

BAT is a metabolically active organ that oxidizes glucose and lipids to drive uncoupled oxidative phosphorylation (mitochondrial respiration), thereby contributing disproportionately to whole-body energy expenditure, despite its relatively small mass (38). Neural circuits in the hypothalamus and brainstem monitor thermal and energetic status within the body, and project to sympathetic premotor neurons that innervate BAT. As such, the hypothalamus aligns thermogenesis to feeding behaviour, circadian rhythms, and stress responses to maintain systemic energy homeostasis (39). Disruption of these neural circuits and/or BAT recruitment associates with weight gain and the development of obesity, insulin resistance, and impaired metabolic flexibility. Conversely, BAT activation and enhanced function improve insulin sensitivity and lipid profile and reduces ectopic fat deposition (40).

Through UCP1-dependent thermogenesis, BAT converts chemical energy into heat, buffering fluctuations in energy balance and improving systemic glucose and lipid homeostasis, particularly under conditions of nutrient excess (41). UCP1 is an inner mitochondrial membrane carrier that dissipates the ‘proton motive force’ by allowing proton re-entry into the mitochondrial matrix without the concomitant synthesis of ATP synthesis. High-resolution structural and biophysical studies reveal how fatty acids activate UCP1 and how purine nucleotides stabilize its inactive conformation, refining our classic model of UCP1-induced mitochondrial proton leakage (6, 42). UCP1 is central to non-shivering thermogenesis. However, multiple UCP1-independent pathways (including creatine and lipid futile cycles and sarco/endoplasmic reticulum Ca^2+^ cycling), also contribute to energy dissipation in brown and beige adipocytes. These parallel mechanisms may compensate when UCP1 levels within BAT are low or absent and are increasingly recognized as potential adjunctive therapeutic targets to enhance total thermogenic output (43, 44).

At the cellular level, BAT activation occurs through β-adrenergic signalling (driven by activity within the sympathetic nervous system [SNS]), that in turn is controlled by the hypothalamic thermoregulatory centre. Norepinephrine released from SNS terminals binds to β-adrenergic receptors on brown adipocytes, thereby inducing intra-cellular pathways that implicate ‘cyclic adenosine monophosphate’ (cAMP) and ‘protein kinase A’ (PKA). This cascade enhances lipolysis and the activation of UCP1, thereby enabling mitochondrial proton leakage and heat generation. β-adrenergic signalling within BAT also promotes transcriptional programs controlled by ‘peroxisome proliferator-activated receptor-gamma coactivator 1-alpha’ (PGC-1α), ‘positive regulatory domain containing 16’ (PRDM16), and other co-regulators that enhance mitochondrial biogenesis and UCP1 expression (41, 45). SNS-mediated BAT activation is essential for adaptive thermogenesis and contributes to systemic metabolic regulation (46–48). Activation of UCP1 by SNS stimulation increases energy expenditure and enhances substrate oxidation, including glucose uptake, linking thermogenesis to improved overall metabolic health (49).

Cold exposure (mediated through enhanced noradrenergic tone) is the most potent physiological stimulus for BAT activation. Cold exposure associates with increased substrate (glucose and lipid) uptake, enhanced insulin sensitivity (through increased glucose transporter 1 [GLUT1] and glucose transporter 4 [GLUT4] translocation), thermogenic capacity (50). Cold exposure also promotes beige adipocyte formation and browning of WAT depots to further optimise thermogenic potential and glucose homeostasis (46, 47, 51). These adaptive responses to cold exposure integrate with endocrine and nutrient sensing pathways (implicating FGF21, irisin, thyroid hormones, and bile acids), thereby further potentiating BAT recruitment and UCP1 expression, aligning energy balance with diverse environmental cues and endocrine signalling (51–53). Beyond cold exposure, dietary and nutritional factors that include polyphenols, capsaicin, and omega-3 fatty acids also enhance BAT thermogenic capacity and heat production by modulating adrenergic and endocrine pathways as well as with the help of Sympathetic Nervous System (SNS) (54). In this regard, mechanisms of BAT activation (and heat production) in response to cold exposure and nutritional factors via SNS are outlined in **Figure 1**.

**Figure 1:**
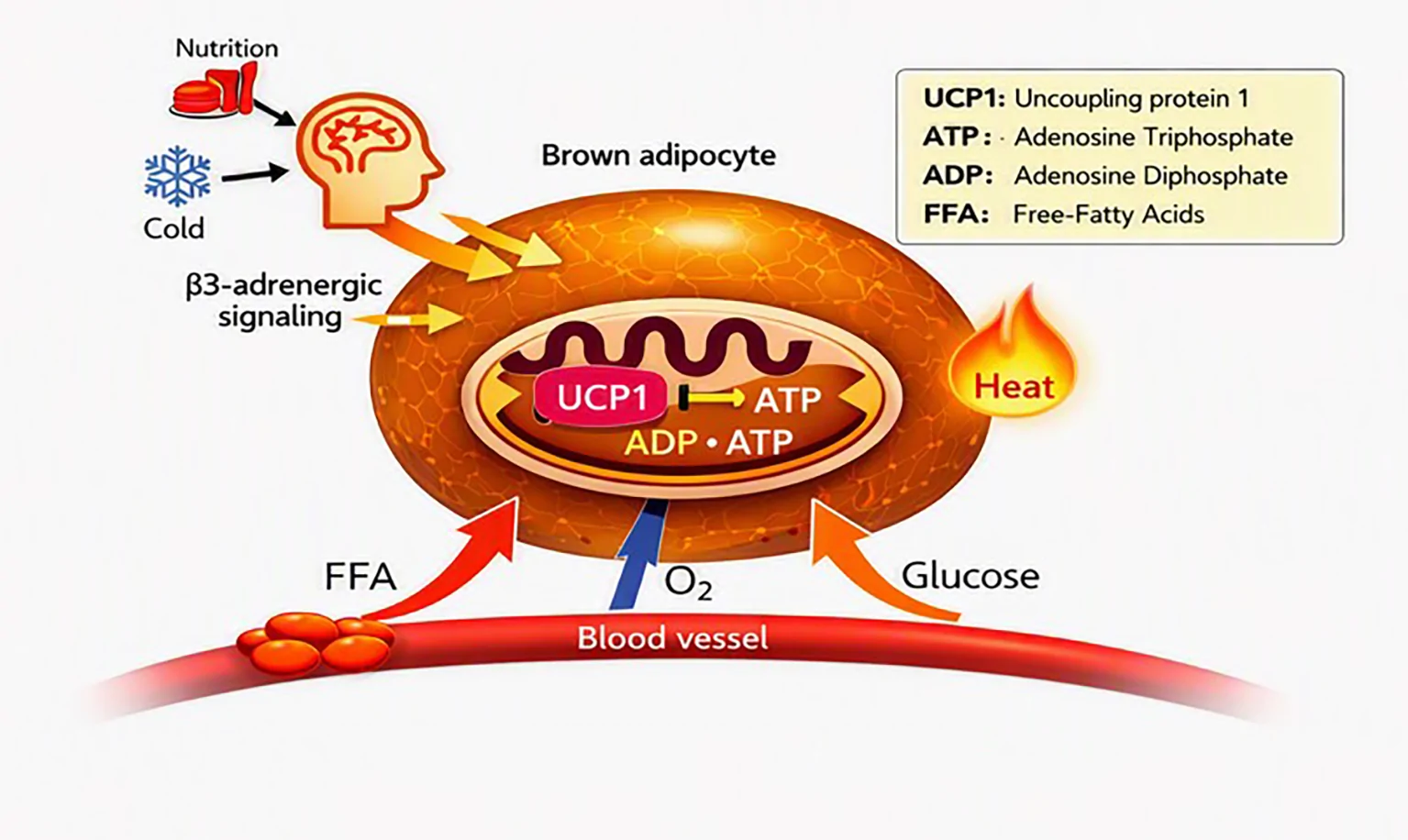
Mechanisms linking nutrition and environmental temperature with activation of brown adipose tissue.

## The role of batokines in metabolic regulation

Key secreted factors from BAT: Batokines are bioactive peptides released from BAT (in response to thermogenic activation from SNS stimulation), including proteins, metabolites and other signalling molecules. Batokines act locally (autocrine/paracrine effects) and systemically (endocrine effects) to influence metabolic homeostasis (43). These secreted factors are derived from the transcriptome and proteome of brown adipocytes (13).

Batokine-mediated regulation of metabolism: Rodent-based data reveal that batokines like PLTP, FGF21, IL-6 and BATSP1 modulate multiple metabolic pathways, including glucose and lipid metabolism. PLTP enhances glucose tolerance, insulin sensitivity, and lipid homeostasis via bile acid release and other hepatic effects. FGF21 (originally identified as a hormone of hepatic origin) regulates lipid oxidation, glucose uptake, and energy expenditure in peripheral tissues, and is implicated as an important regulator of systemic glucose and lipid metabolism (55). IL-6 also contributes towards systemic energy balance. Finally, BATSP1 promotes adipocyte thermogenesis and improve systemic glucose metabolism (56, 57).

Endocrine effects of batokines linking BAT with the periphery: Emerging research highlights bidirectional communications between BAT and peripheral organs such as the liver, skeletal muscle, and pancreas. Batokines like FGF21, IL-6, EPDR1, NRG4 and PLTP may influence pancreatic β-cell function and insulin secretion, suggesting the existence of a BAT-islet endocrine axis that influences glucose homeostasis. Batokines like NRG4 also inhibit *de novo* lipogenesis and enhance fatty acid oxidation within the liver, demonstrating crosstalk between BAT and the liver (11, 58). CXCL14 contributes to immune modulation and tissue recruitment, suggesting roles in immune and metabolic integration. Whilst the precise molecular mechanisms underlying these endocrine pathways are still incompletely understood, the existing data reveal that BAT communicates with peripheral organs and integrates systemic metabolic responses (59).

Batokine effects on glucose metabolism: Several batokines influence glucose metabolism through a variety of mechanisms, including improved insulin sensitivity, glucose uptake into peripheral tissues, and β-cell insulin release.

- *FGF21* enhances insulin sensitivity and glucose uptake into adipose tissue and skeletal muscle through BAT dependent and -independent pathways, reflecting coordinated endocrine and thermogenic regulation (60). FGF21 also potentiates BAT activation and thermogenic output (through enhanced UCP1 expression and mitochondrial biogenesis) via its synergy with UCP1 actions (61).
- *BATSP1*, like FGF21, also directly modulates UCP1 expression and BAT thermogenic activity and facilitates improved glucose homeostasis in ‘diet-induced obesity’ rodent models (62, 63). Mechanistically, BATSP1 reduces ‘forkhead box protein 01’ (FOXO1) suppression of UCP1 within BAT, thereby facilitating increased UCP1 transcription within BAT and enhanced energy expenditure. BATSP1 also promotes the ‘browning’ of WAT and illustrates how batokines can amplify UCP1-dependent thermogenesis beyond classical BAT (57, 58).
- *PLTP* is a further batokine implicated in the control of glucose metabolism. PLTP improves glucose tolerance and systemic insulin responses, possibly through the modulation of lipid metabolism and bile acid signalling. Through its interactions with liver and skeletal muscle, PLTP complements the effects of UCP1-mediated thermogenesis in improving whole-body glucose homeostasis (62, 64).
- *IL-6* illustrates a dual role of adipokines and batokines in inflammation and metabolism. IL-6 is both an adipokine and batokine. In the context of obesity, IL-6 as an adipokine and released from excessive WAT contributes towards obesity-associated low-grade inflammation, hepatic insulin resistance, and the hepatic production of glucose. Conversely, when it is released as a batokine from activated BAT during thermogenic activation, IL-6 facilitates increased glucose uptake by BAT and improved systemic insulin sensitivity (65). IL-6 as a batokine may also modulate UCP1 expression indirectly by enhancing SNS drive to BAT (64). Therefore, some batokines like IL-6 appear to have dual functions, and the cellular and metabolic responses to their release appears contingent upon context (including environmental, metabolic and stress-related preparedness).
- *NRG4* attenuates hepatic lipogenesis and may enhance glucose uptake into peripheral tissues, thereby providing supportive systemic metabolic effects that integrate with UCP1 activation (65). Whilst mechanistic links between UCP1 signalling and NRG4 expression remain under investigation, emerging evidence supports their coordinated roles in metabolic homeostasis (66).
- *GDF15* is upregulated in response to metabolic stress and may modulate energy expenditure and glucose utilization through both central and peripheral pathways, although its direct effect on UCP1 transcription is less clear (64, 67).

## BAT dysfunction in obesity and T2D

Having provided an overview of the physiology of BAT, and the diverse role of batokines in the regulation of metabolism, we now discuss how BAT dysfunction contributes towards weight gain and the development of obesity, and its associated metabolic dysfunction, including T2D. Given the metabolic benefits of activated BAT, it is perhaps not surprising that obesity and T2D associate with a reduction in BAT mass and activity, thereby contributing to impaired metabolic flexibility, dysregulated glucose homeostasis, and diminished lipid clearance. BAT dysfunction in obesity stems primarily from low-grade chronic inflammation and extracellular matrix remodeling, wherein macrophage-derived cytokines (such as TNF-α, IL-1β, and TGF-β) drive fibrosis, reduced vascularization, and impaired adrenergic responsiveness. Concurrently, mitochondrial stress with reduced UCP1 function, diminished oxidative capacity, and altered lipid handling further suppresses thermogenesis and drives BAT dysfunction with the adoption of a WAT-like phenotype (68).

Understanding the mechanisms that implicate BAT dysfunction in the development of metabolic disorders, and how this process can be reversed, has become a major research focus. In this section, we discuss the known mechanisms that down-regulate BAT activation in obesity and T2D, and the impact of BAT dysfunction on obesity and T2D.

### Mechanisms that down-regulate BAT activation in obesity and T2D

BAT activation is regulated by SNS signalling, mitochondrial biogenesis, batokines, and nutrient-derived signals (69). As outlined above, BAT contributes to systemic insulin sensitivity by clearing glucose and triglycerides from the circulation. In obesity and T2D, chronic low-grade inflammation, hyperinsulinemia, and elevated levels of serum triglycerides impair β-adrenergic signalling from the SNS and thereby reduce UCP1 expression within BAT (70). Obesity-associated fibrosis and mitochondrial dysfunction within BAT further limit its thermogenic capacity (71, 72). BAT fibrosis in obesity and type 2 diabetes arises largely from chronic inflammation and extracellular-matrix remodelling driven by macrophage infiltration, TGF-β signalling, and reduced vascularisation, which together promote collagen deposition and impair thermogenic function. Preclinical models consistently show that metabolic stress and low-grade inflammation shift brown adipocytes toward a more fibrotic, dysfunctional phenotype (73).

Furthermore, batokines such as FGF21, NRG4, and IL-6 normally enhance thermogenesis, but their secretion and signalling become dysregulated in the context of metabolic dysfunction (39, 74). These alterations collectively weaken the ability of BAT to respond to cold or pharmacological stimuli (39, 72, 74). Impaired β-adrenergic signalling and BAT fibrosis results in reduced UCP1 activity and thermogenesis within BAT that has multiple down-stream effects that include reduced energy expenditure (exacerbating a positive energy balance) (75), increased lipid accumulation within liver and skeletal muscle further promoting insulin resistance, and reduced secretion of batokines that can further compound insulin resistance and impaired glucose and lipid metabolism. Thus, in the scenario of obesity and T2D, BAT dysfunction and insulin resistance reinforce each other in a vicious and detrimental metabolic circle (74).

### BAT dysfunction and its impact on obesity

Obesity is characterized by reduced BAT mass and activity (through diminished mitochondrial and UCP1 content), and impaired ‘browning’ capacity of WAT. These changes lower basal metabolic rate and decrease adaptive thermogenesis, thereby promoting further weight gain (71). Furthermore, obesity-associated adipocyte hypertrophy limits vascularization of BAT and it supply of oxygen and nutrients, thereby contributing to a whitening of BAT (76). As BAT becomes less functional, its ability to oxidize fatty acids declines, contributing towards elevated serum levels of glucose and triglycerides and worsened metabolic outcomes (77).

### BAT dysfunction and its impact on T2D

Individuals with T2D have significantly reduced glucose uptake into and diminished thermogenic activity within BAT. Insulin resistance blunts insulin stimulated glucose transport into brown adipocytes, thereby limiting the availability of glucose as a substrate for BAT thermogenesis (78). Diabetic neuropathy, a long-term microvascular complication of T2D, may further impair innervation of the SNS within BAT, thereby reducing its capacity for norepinephrine-mediated activation. Moreover, hyperglycemia and lipotoxicity induce oxidative stress and mitochondrial damage within BAT, further suppressing UCP1 expression. Clinical imaging studies demonstrate consistently lower BAT volume and activity in people with T2D compared with healthy euglycemic individuals (79). Finally, BAT-secreted batokines may play a dual role in the risk of T2M development. In this regard, studies have shown that a group of these BAT biomolecules are involved in the pathogenesis of T2D, and another group is involved in its protection (80).

## The therapeutic potential of BAT activation within vital organs

There is potential for therapeutic approaches that activate BAT as a novel therapeutic approach in the management of obesity and T2D (outlined in **Table 1**). Broadly, these therapeutic strategies straddle both lifestyle and pharmacotherapies. Cold exposure remains the most potent physiological activator of BAT. Repeated mild cold exposure increases BAT volume, enhances UCP1 expression, and improves insulin sensitivity even in people with obesity (81). Therefore, lifestyle interventions such as controlled cold exposure and physical exercise represent one approach to drive thermogenesis within BAT, as a modifiable component of human energy expenditure (38), 40). Other lifestyle interventions include nutritional strategies. Nutrients like capsaicin, catechins, resveratrol, and ‘omega-3 fatty acids’, can stimulate BAT-mediated thermogenesis through ‘Transient Receptor Potential’ (TRP) channels or mitochondrial pathways (82, 83). Intermittent fasting and ketogenic diets can also enhance BAT activity by increasing sympathetic tone and promoting fatty acid oxidation (84) (Figure). Unfortunately, there is reduced efficacy of these outlined lifestyle interventions in obesity due to impaired SNS signalling and reduced BAT responsiveness (77).

**Table 1:** Therapeutic potential of BAT activation in obesity and T2D.

Therapeutic effect	Mechanistic basis	Evidence type
Improved glucose disposal	Increased uptake of glucose via insulin-independent and insulin-sensitising pathways	Human, Animal
Enhanced insulin sensitivity	UCP1-mediated thermogenesis reduces circulating lipids and inflammatory cytokines	Human, Animal
Reduced hepatic glucose output	BAT activation lowers gluconeogenesis through improved systemic metabolic signalling	Animal
Improved lipid metabolism	Increased fatty acid oxidation and clearance of triglyceride-rich lipoproteins	Human, Animal
Lower systemic inflammation	BAT-derived batokines reduce pro-inflammatory signalling associated with obesity	Animal, *In vitro*
Increased whole-body energy expenditure	Cold-induced or pharmacological thermogenesis elevates caloric burn	Human, Animal
Cardiometabolic protection	BAT activation improves vascular function and reduces lipid burden	Animal
Improved postprandial glycemia	Enhanced glucose uptake after meals via thermogenic activation	Human
Reduction in ectopic fat accumulation	Increased oxidation of circulating fatty acids reduces hepatic and cardiac steatosis	Animal
Hormonal/batokine signalling benefits	BAT-secreted factors (e.g., 12,13-diHOME) improve metabolic flexibility	Animal, *In vitro*

UCP1, Uncoupling Protein 1; BAT, Brown Adipose Tissue; 12,13 diHOME, 12,13-dihydroxy-9Z-octadenoic acid.

Beyond lifestyle approaches, GLP-1 based therapies, including semaglutide and dual incretin agents such as tirzepatide, enhance BAT metabolic activity by improving thermogenic gene expression and reducing inflammatory signalling in brown adipocytes. Tirzepatide promotes BAT thermogenesis and upregulates batokines such as Bone Morphogenetic Protein 8B (BMP8B) and CXCL14, contributing to improved systemic energy expenditure and metabolic homeostasis. These incretin-related mechanisms highlight BAT as a therapeutically relevant organ that could mediate some of the metabolic effects of GLP-1 based therapies (including glucose regulation, lipid handling, and cardiometabolic protection) (85). Emerging evidence shows that GLP-1 based therapies can indirectly enhance BAT oxidative metabolism by improving sympathetic drive and mitochondrial efficiency, thereby supporting whole body energy expenditure (86). Negative regulators of BAT repressors also represent an interesting therapeutic approach that could enhance BAT activity and energy expenditure. Several batokines exert down-regulating effects on whole body metabolic homeostasis and, specifically on BAT activity. Negatively regulating these BAT repressors such as through monoclonal antibodies or small synthetic molecules may help to restore BAT function, which is often impaired in obesity. Recently, two batokines have been identified as BAT repressors whose BAT specific ablation enhances thermogenic activity and whole-body metabolic health: Acyl-CoA-Binding Protein (ACBP) (87). and Cardiotrophin-like cytokine factor 1 (CLCF1) (88).

Beyond GLP-1 based therapies and negative regulators of BAT repressors, novel pharmacotherapies could mimic the cold-induced adrenergic activation of BAT through the targeting of mitochondrial uncoupling, nutrient sensing or batokine signalling (40, 50). Such synthetic ‘mitochondrial uncouplers’ aim to mimic UCP1-mediated proton leakage in a tissue and dose-selective manner. Through enhancing UCP1-dependent thermogenesis in BAT, these therapies would improve glucose and lipid metabolism, insulin sensitivity, and obesity-associated inflammation. These are key drivers of cardiac, renal, and hepatic dysfunction (89). BAT activation improves glycemic control by increasing glucose disposal, enhancing insulin sensitivity, and reducing hepatic glucose output (90). Clinical studies show that individuals with higher BAT activity have lower fasting glucose and improved HbA1c levels (91). Furthermore, BAT stimulation increases whole body glucose uptake and reduces postprandial glycemia (79).

The protonophore, ‘N^5^, N^6^-bis (2-fluorophenyl) [1,2,5] oxadiazolo [3,4-b] pyrazine-5,6-diamine’ (BAM15), is a mitochondrial uncoupler that has shown therapeutic potential in preclinical models of obesity and insulin resistance. Other molecules like FGF21 analogs, and ‘Peroxisome Proliferator-Activated Receptor γ’ (PPARγ) modulators have demonstrated potential for enhanced BAT activation (75). β3 adrenergic agonists like Mirabegron can increase glucose uptake and energy expenditure within BAT in humans. However, Mirabegron has off-target cardiovascular side effects that limit its widespread use (78). In a future scenario, combining intermittent cold exposure with novel pharmacological approaches may yield synergistic therapeutic effects of BAT activation (79).

For all their therapeutic benefits, there remain key challenges for mitochondrial uncouplers that target BAT directly. These include over-compensated enhancement of appetite and food intake in response to BAT activation and increased energy expenditure, thereby offsetting any additional burnt calories. Consequently, BAT-induced thermogenesis (through mitochondrial uncoupling or other mechanisms) rarely leads to significant or sustained weight loss, as natural homeostatic mechanisms restore energy balance (92).

A further challenge is the importance of BAT selectivity and avoiding off-target effects that can induce mitochondrial stress in non-BAT tissues. These challenges provide a rationale for the development of molecules that, rather than targeting BAT directly, optimize BAT activity *indirectly*, thereby avoiding unwanted off-target side effects of SNS activation. These molecules integrate with endogenous UCP1 and its control through the batokine network (BAT-derived signalling pathways) (93). Therapeutically targeting natural batokines helps to avoid the adverse off-target effects (such as activation of the SNS) that can occur with direct BAT enhancement (94). Through targeting batokines, this provides a broader range of benefits than direct BAT activation, extending to diverse organ-specific effects. Indeed, optimizing BAT function through mimicking batokine signalling may represent a novel adjunctive therapy for heart failure and metabolic syndrome, conditions characterized by impaired energy homeostasis (95).

In the rest of this section, we discuss the diverse therapeutic potential of BAT activation (including through batokines) within vital organs: heart, lung, kidney, and liver (outlined in **Table 2**).

**Table 2:** Therapeutic effects of BAT activation in vital organs.

Vital organs	Therapeutic effects of BAT activation in vital organs
Heart	Improving calcium cycling and mitochondrial function in relation to cardiac contraction Improving lipid and glucose profiles in relation to reduced atherosclerosis Reducing pathological stress responses and improving cardiac resilience Acceleration of beneficial metabolic regeneration in the heart Improving myocardial energy metabolism Improving cardiac perfusion
Lung	Improved lung immune responses and metabolic stress Modulation of the inflammatory response Support for anti-inflammatory networks Increased systemic immune response Modulation of oxidative stress
Kidney	Reducing metabolic stress that contributes to the progression of chronic kidney disease Improving inflammation and oxidative stress in the kidney Optimizing renal glucose management Improving renal fibrosis Improving albuminuria
Liver	Protecting the liver against hepatic steatosis and inflammatory damage Improving lipid absorption and lipoprotein processing in the liver Decreased lipogenesis and ferroptosis in the liver Important role in liver cleansing Improving insulin sensitivity
Biliary	Improving systemic glucose transport and energy homeostasis through bile acid signalling Increases the hepatic conversion of cholesterol into bile acids Linking thermogenic fat metabolism to bile acid synthesis Improving bile acid homeostasis and bile duct function

BAT, Brown Adipose Tissue.

### Cardiac effects of BAT activation

BAT activation exerts cardioprotective effects through improved myocardial energy metabolism and limitation of pathological remodelling through UCP1-dependent thermogenesis and endocrine signalling (96, 97). BAT secretes endocrine factors that modulate cardiac metabolism and protect heart function. For example, BAT-derived FGF21 improves myocardial resilience against ischemia/reperfusion injury and supports cardiomyocyte survival (98). BAT also releases 12,13-diHOME, which enhances cardiac contractility and metabolic efficiency by improving calcium cycling and mitochondrial function (99). These batokines highlight endocrine crosstalk between BAT activation and cardiovascular health (100). Activation of BAT thermogenesis via UCP1 reduces early cardiac hypertrophic and profibrotic signalling in models of pressure overload, thereby accelerating beneficial metabolic remodelling in the heart (97). Furthermore, cold-induced BAT activation associates with improved cardiac resilience by attenuating the pathological stress responses (100). Indirectly, BAT activation also improves cardiac health and function through reduced overall cardiovascular risk (including improved lipid and glucose profiles, and reduced atherosclerosis) (101). (Conversely, BAT dysfunction associates with exacerbated myocardial injury and adverse remodelling, emphasizing its cardioprotective role) (102).

### Pulmonary effects of BAT activation

Metabolic dysregulation (including insulin resistance and dyslipidaemia) is a key component of chronic lung diseases, in which systemic inflammation and altered adipokine profiles contribute towards lung dysfunction and associated metabolic disorders (103, 104). Adipokines such as leptin and adiponectin mediate the pathophysiology of inflammatory lung conditions like asthma and COPD, linking metabolism to lung pathology (105). Emerging evidence supports a role for batokines in the systemic immune response and modulation of inflammatory tone beyond the metabolic organs, potentially impacting pulmonary inflammation and overall energy homeostasis through endocrine signalling (106). Within the lung, batokines modulate inflammatory responses and oxidative stress, suggesting indirect protection from pulmonary metabolic injury (107). BAT activation, through increasing energy expenditure and altering substrate availability, may also have indirect effects on lung immune responses and metabolic stress (63). Furthermore, batokines interact with immune cells and support anti-inflammatory networks that could influence inflammation and metabolic adaptations within the lung (108).

### Renal effects of BAT activation

BAT activation influences renal physiology (including renal glucose handling and oxidative stress) through improved insulin sensitivity and lipid handling. BAT activation reduces metabolic stress that contributes towards the progression of chronic kidney disease (109) and thereby ameliorates key pathological features of ‘diabetic kidney disease’ (DKD), including albuminuria, renal fibrosis, inflammation, and oxidative stress, through metabolic improvements and enhanced adipokine/miRNA signalling (110). Batokines modulate renal cell survival under metabolic stress. One example is BAT-derived NRG4 that prevents glucose-induced podocyte apoptosis in diabetic nephropathy, highlighting a protective metabolic interaction (66). BAT activation also reduces renal oxidative stress and albuminuria via enhanced adipokine signalling and ‘5’ adenosine monophosphate-activated protein kinase’ (AMPK) and ‘Sirtuin 1’ (Sirt1) pathways in diabetic rodent models (110). Through improved systemic glucose metabolism, batokines may also optimize renal glucose handling indirectly (64). Finally, in rodent models of DKD, BAT transplantation reactivates endogenous BAT and mitigates against DKD progression through suppression of fibrotic regulators and enhancement of protective factors like ‘microRNA-30b’ (miR-30b) (66).

### Hepatic effects of BAT activation

In the liver, batokines like NRG4 suppress lipogenesis and ferroptosis, thereby protecting against hepatic steatosis and inflammatory injury and improving insulin sensitivity (97). (Mechanisms implicate the hepatic lipogenic signalling pathways ‘Liver X Receptor’ [LXR] and ‘Sterol Regulatory Element-Binding Protein 1c’ [SREBP-1c]) (11). Furthermore, batokine-mediated lipid uptake and lipoprotein processing enhance hepatic clearance of triglyceride remnants, thereby improving plasma lipid profile (101). NRG4 protects against liver injury by suppressing ferroptosis in models of sepsis, highlighting crosstalk between BAT and hepatic pathology (66, 111). Moreover, BAT activation modulates lipid flux and whole-body metabolism, which may indirectly influence renal and metabolic disease progression (112).

Emerging evidence also reveals that BAT-mediated metabolic regulation may affect bile acid homeostasis and bile duct function via a ‘liver-adipose’ crosstalk, linking BAT activity to hepatobiliary health (113). Cold induced activation of BAT enhances the hepatic conversion of cholesterol into bile acids, thereby linking thermogenic lipid metabolism with bile acid synthesis (114). Bile acids mediate endocrine signalling through binding to the ‘Takeda G-protein coupled receptor 5’ (TGR5) in BAT, enhancing UCP1-dependent thermogenesis through increased levels of intracellular cAMP and potentiating the effects of thyroid hormone on BAT activation (115, 116). Such bile acid–TGR5 signalling also improves systemic glucose handing and energy homeostasis (117). (There are differential effects of bile acid receptors within BAT, with ‘Farnesoid X receptor’ [FXR] signalling having suppressive effects on thermogenic gene expression within BAT) (118). The diverse mechanisms outlined here highlight the endocrine influence of BAT on the metabolic health of the liver (119) and underlie an inverse correlation between BAT activity and the severity of ‘Metabolic Dysfunction-Associated Steatotic Liver Disease’ (MASLD) (120).

## Discussion

Our understanding of BAT activation remains challenging and incomplete due to the variability of BAT mass, its natural state of activation, metabolic responsiveness, and limited imaging precision of BAT in humans (38). It is important for future studies to explore the interactions between UCP1 activation and batokine release, and their therapeutic coordination to target metabolic disease safely. In this endeavour, we need to address any concerns regarding the chronic activation of BAT, with its potential for excessive energy expenditure, cardiovascular strain, and unintended thermogenic overstimulation (121). Longer term stimulation of BAT-related UCP1 activity may also disrupt the systemic metabolic balance and increase oxidative stress in susceptible individuals (122). Moreover, chronic batokine secretion (either physiologically or therapeutically induced) could adversely affect endocrine signalling and impair organ homeostasis if not regulated properly (58). Future novel therapies should balance BAT activation with proper safety monitoring to avoid any potential adverse metabolic and/or cardiovascular outcomes (123).

A key challenge in this field relates to the physiological differences of WAT and BAT regulation between animals (specifically rodents) and humans (124). These species-level discrepancies stymie our interpretation of batokine function, its systemic metabolic effects across species, and its therapeutic potential in humans (125). Furthermore, the environmental and behavioral factors known to influence BAT activation in humans are difficult to replicate in controlled animal-based studies (126). Therefore, improved models that more accurately reflect BAT physiology in humans are essential to evaluate the therapeutic potential of UCP1–batokine interactions (127). Personalized metabolic novel therapies may emerge by integrating individual variabilities in BAT mass and activity, UCP1 expression, and batokine secretion profiles (128). Individual genetic and epigenetic determinants that influence thermogenic responsiveness of BAT could also guide tailored interventions for the future management of obesity and T2D (125). Moreover, advances in BAT imaging and multi-omics molecular profiling would enable precise characterization and stratification of patient-specific metabolic phenotypes (124), thereby facilitating and enabling an individualized approach to the modulation of UCP1–batokine pathways, to optimize therapeutic efficacy and minimize adverse effects (129, 130). In this endeavour, combining future therapies that activate UCP1 with batokine-based interventions (including novel molecular targets implicated in pathways that control adipocyte differentiation and mitochondrial remodelling) may offer synergistic therapeutic effects that further maximize systemic benefits whilst minimizing safety risks (131). Finally, future gene-based strategies that target UCP1 expression and upstream regulators within BAT are emerging as powerful tools to enhance BAT thermogenesis and modulate batokine signalling (132).

To summarize, the BAT-batokine axis remains under-explored and under-utilized therapeutically. Obesity, T2D and other obesity-related dysmetabolic conditions (including those like chronic respiratory conditions that whilst not considered metabolic in origin, nonetheless have important metabolic aspects to pathophysiology) represent a major contributor to premature mortality, morbidity, and healthcare expenditure globally. There are limitations of our current therapeutic options when considered from the perspective of the vast therapeutic potential of the BAT-batokine axis. Such novel therapies would transform our management of obesity and T2D, and multiple other chronic conditions. Rather than simply providing a mechanism for weight loss (through negative energy balance) or improving glycaemic control, such novel therapies would impart diverse and holistic therapeutic benefits on key organs essential for metabolic efficiency and flexibility. The BAT-batokine axis has finally come of age. Now is their time in the therapeutic limelight.

## Glossary

BAT: Brown Adipose Tissue
WAT: White Adipose Tissue
T2D: Type 2 Diabetes Mellitus
GLP-1: Glucagon-like Peptide-1
SNS: Systemic Nervous System
UCP1: Uncoupling Protein 1
ATP: Adenosine Triphosphate
FGF21: Fibroblast Growth Factor-21
IL-6: Interleukin-6
NRG4: Neuregulin-4
EPDR1: Ependymid Related Protein 1
PLTP: Phospholipid Transfer Protein
BATSP1: BAT-secreted peptide 1
GDF15: Growth Differentiation Factor 15
12,13 diHOME: 12,13-dihydroxy-9Z-octadenoic acid
CXCL14: C-X-C motif chemokine ligand 14
cAMP: cyclic adenosine monophosphate
PKA: protein kinase A
PGC-1α: peroxisome proliferator-activated receptor-gamma coactivator 1-alpha
PRDM16: positive regulatory domain containing 16
GLUT1: glucose transporter 1
GLUT4: glucose transporter 4
FOXO1: forkhead box protein 01
TNF-α: tumor necrosis factor alpha
TGF-β: transforming growth factor beta
TRP: Transient Receptor Potential
BMP8B: Bone Morphogenetic Protein 8B
ACBP: Acyl-CoA-Binding Protein
CLCF1: Cardiotrophin-like cytokine factor 1
HbA1c: hemoglobin A1c
BAM15: pyrazine-5,6-diamine’
PPARγ: Peroxisome Proliferator-Activated Receptor γ
DKD: diabetic kidney disease
AMPK: adenosine monophosphate-activated protein kinase
Sirt1: Sirtuin 1
miR-30b: microRNA-30b
SREBP-1c: Sterol Regulatory Element-Binding Protein 1c
LXR: Liver X Receptor
TGR5: Takeda G-protein coupled receptor 5
FXR: Farnesoid X receptor
MASLD: Metabolic Dysfunction-Associated Steatotic Liver Disease.

## References

[1] Kajimura S Seale P . Brown and beige fat: physiological roles beyond heat generation. Cell Metab. (2015) 22:546–59. doi: 10.1016/j.cmet.2015.09.007 26445512 PMC4613812

[2] Spiegelman BM . Transcriptional control of mitochondrial energy metabolism through the PGC1 coactivators. Novartis Found Symp. (2007) 287:60–3. doi: 10.1002/9780470725207.ch5 18074631. discussion 63-9.

[3] Su S Guntur AR Nguyen DC Fakory SS Doucette CC Leech C . A renewable source of human beige adipocytes for development of therapies to treat metabolic syndrome. Cell Rep. (2018) 25:3215–28. doi: 10.1016/j.celrep.2018.11.037 30540952 PMC6375695

[4] Scheele C Wolfrum P . Brown adipose crosstalk in tissue plasticity and human metabolism. Endocr Rev. (2020) 41:53–65. doi: 10.1210/endrev/bnz007 31638161 PMC7006230

[5] Li Y Fromme T Klingenspor M . Meaningful respirometric measurements of UCP1-mediated thermogenesis. Biochimie. (2017) 134:56–61. doi: 10.1016/j.biochi.2016.12.005 27986537

[6] Jones SA Ruprecht JJ Crichton PG Kunji ERS . Structural mechanisms of mitochondrial uncoupling protein 1 regulation in thermogenesis. Trends Biochem Sci. (2024) 49:506–19. doi: 10.1016/j.tibs.2024.03.005 38565497

[7] Ježek P Jabůrek M Porter RK . Uncoupling mechanism and redox regulation of mitochondrial uncoupling protein 1 (UCP1). Biochim Biophys Acta Bioenerg. (2019) 1860:259–69. doi: 10.1016/j.bbabio.2018.11.007 30414927

[8] Seale P Kajimura S Spiegelman BM . Transcriptional control of brown adipocyte development and physiological function of mice and men. Genes Dev. (2009) 23:788–97. doi: 10.1101/gad.1779209 19339685 PMC2763499

[9] Kozak LP . Brown fat and the myth of diet-induced thermogenesis. Cell Metab. (2010) 11:263–7. doi: 10.1016/j.cmet.2010.03.009 20374958 PMC2867325

[10] Nedergaard J Cannon B . UCP1 mRNA does not produce heat. Biochim Biophys Acta. (2013) 1831:943–9. doi: 10.1016/j.bbalip.2013.01.009 23353596

[11] Wang G-X Zhao X-Y Meng Z-X Kern M Dietrich A Chen Z . The brown fat-enriched secreted factor Nrg4 preserves metabolic homeostasis through attenuation of hepatic lipogenesis. Nat Med. (2014) 20:1436–43. doi: 10.1038/nm.3713 25401691 PMC4257907

[12] Lynes KE Tseng Y-H Cohen P . The cold-induced lipokine 12,13-diHOME promotes fatty acid transport into brown adipose tissue. Nat Med. (2017) 23:631–7. doi: 10.1038/nm.4297 28346411 PMC5699924

[13] Ghesmati Z Rashid M Fayezi S Gieseler F Alizadeh E Darabi M . An update on the secretory functions of brown, white, and beige adipose tissue: towards therapeutic applications. (2023) 25:279–308. doi: 10.1007/s11154-023-09850-0 PMC10942928 38051471

[14] Martins FF Martins BC Teixeira AVS Ajackson M Souza-Mello V Daleprane JB . Brown adipose tissue, batokines, and bioactive compounds in foods: an update. Mol Nutr Food Res. (2024) 68:e2300634. doi: 10.1002/mnfr.202300634 38402434

[15] Wang G-X Zhao X-Y Lin JD . The brown fat secretome: metabolic functions beyond thermogenesis. Trends Endocrinol Metab. (2015) 26:231–7. doi: 10.1016/j.tem.2015.03.002 25843910 PMC4417028

[16] Wagner L Estrada-Kunz J Roth L Weiner J Kralisch S Hoffmann A . The impact of neuregulin 4 on metabolic dysregulation in lipodystrophy. Endocrinology. (2025) 166:bqaf112. doi: 10.1210/endocr/bqaf112 40580113 PMC12236340

[17] May W Goedecke JH Conradie-Smit M . The science of obesity. S Afr Med J. (2025) 115:e3600. doi: 10.7196/SAMJ.2025.v115i9b.3600 41378649

[18] Khanna S Mann G Bhat A Lin A Patel A Nicholls SJ . Role of systemic and epicardial adipose tissue in cardiometabolic disease. Nat Rev Cardiol. (2026). doi: 10.1038/s41569-026-01304-9 42303809

[19] Ma Y Gao M Liu D . Preventing high fat diet-induced obesity and improving insulin sensitivity through neuregulin 4 gene transfer. Sci Rep. (2016) 6:26242. doi: 10.1038/srep26242 27184920 PMC4869101

[20] Pereira RO Abel ED . BATokines in metabolic liver disease: good cops or bad cops? EMBO J. (2024) 43:4807–9. doi: 10.1038/s44318-024-00239-6 39322755 PMC11535238

[21] Fenzl A Kiefer FW . Brown adipose tissue and thermogenesis. Horm Mol Biol Clin Investig. (2014) 19:25–37. doi: 10.1515/hmbci-2014-0022 25390014

[22] Shapira SN Seale P . Transcriptional control of brown and beige fat development and function. Obes (Silver Spring). (2019) 27:13–21. doi: 10.1002/oby.22334 30569639 PMC6309799

[23] Nishio M Saeki K . The remaining mysteries about brown adipose tissues. Cells. (2020) 9:2449. doi: 10.3390/cells9112449 33182625 PMC7696203

[24] Yasuda T Masaki T Sakata T Yoshimatsu H . Hypothalamic neuronal histamine regulates sympathetic nerve activity and expression of uncoupling protein 1 mRNA in brown adipose tissue in rats. Neuroscience. (2004) 125:535–40. doi: 10.1016/j.neuroscience.2003.11.039 15099666

[25] Cypess AM Lehman S Williams G Tal I Rodman D Goldfine AB . Identification and importance of brown adipose tissue in adult humans. N Engl J Med. (2009) 360:1509–17. doi: 10.1056/NEJMoa0810780 19357406 PMC2859951

[26] Verkerke ARP Wang D Yoshida N Taxin ZH Shi X Zheng S . BCAA-nitrogen flux in brown fat controls metabolic health independent of thermogenesis. Cell. (2024) 187:2359–74.e18. doi: 10.1016/j.cell.2024.03.030 38653240 PMC11145561

[27] Betz MJ Enerbäck S . Human brown adipose tissue: what we have learned so far. Diabetes. (2015) 64:2352–60. doi: 10.2337/db15-0146 26050667

[28] Aldiss P Dellschaft N Sacks H Budge H Symonds ME . Beyond obesity - thermogenic adipocytes and cardiometabolic health. Horm Mol Biol Clin Investig. (2017) 31. doi: 10.1515/hmbci-2017-0007 28593918

[29] Leiria LO Wang C-H Lynes MD Yang K Shamsi F Sato M . 12-Lipoxygenase regulates cold adaptation and glucose metabolism by producing the omega-3 lipid 12-HEPE from brown fat. Cell Metab. (2019) 30:768–83.e7. doi: 10.1016/j.cmet.2019.07.001 31353262 PMC6774888

[30] Chait A den Hartigh LJ . Adipose tissue distribution, inflammation and its metabolic consequences, including diabetes and cardiovascular disease. Front Cardiovasc Med. (2020) 7:22. doi: 10.3389/fcvm.2020.00022 32158768 PMC7052117

[31] van Marken Lichtenbelt WD Vanhommerig JW Smulders NM Drossaerts JMAFL Kemerink GJ Bouvy ND . Cold-activated brown adipose tissue in healthy men. N Engl J Med. (2009) 360:1500–8. doi: 10.1056/NEJMoa0808718 19357405

[32] Cero C Lea HJ Zhu KY Shamsi F Tseng Y-H Cypess AM . β3-Adrenergic receptors regulate human brown/beige adipocyte lipolysis and thermogenesis. JCI Insight. (2021) 6:e139160. doi: 10.1172/jci.insight.139160 34100382 PMC8262278

[33] Betz MJ Enerbäck S . Human brown adipose tissue: what we have learned so far. Diabetes. (2018) 67:1729–37. doi: 10.2337/db15-0146 26050667

[34] Fernández-Verdejo R Marlatt KL Ravussin E Galgani JE . Contribution of brown adipose tissue to human energy metabolism. Mol Aspects Med. (2019) 68:82–9. doi: 10.1016/j.mam.2019.07.003 31306668 PMC7112661

[35] Wang D Li M Lu T Matsushita M Sakai J Saito M . Brown fat protects against hepatic oxidative stress by remodeling the circulating metabolome. Cell Metab. (2026) 14:S1550–4131(26)00246-9. doi: 10.1016/j.cmet.2026.06.020 42447865 PMC13440245

[36] Iqbal M Yu Q Tang J Xiang J . Unraveling the gut microbiota's role in obesity: key metabolites, microbial species, and therapeutic insights. J Bacteriol. (2025) 207:e0047924. doi: 10.1128/jb.00479-24 40183584 PMC12096833

[37] Taniguchi H Hashimoto Y Dowaki N Nirengi S . Association of brown adipose tissue activity with circulating sex hormones and fibroblast growth factor 21 in the follicular and luteal phases in young women. J Physiol Anthropol. (2024) 43:23. doi: 10.1186/s40101-024-00371-6 39354624 PMC11446134

[38] Cypess AM Cannon B Nedergaard J Kazak L Chang DC Krakoff J . Emerging debates and resolutions in brown adipose tissue research. Cell Metab. (2025) 37:12–33. doi: 10.1016/j.cmet.2024.11.002 39644896 PMC11710994

[39] Cohen P Kajimura S . The cellular and functional complexity of thermogenic fat. Nat Rev Mol Cell Biol. (2021) 22:393–409. doi: 10.1038/s41580-021-00350-0 33758402 PMC8159882

[40] van der Lans AAJJ Hoeks J Brans B Vijgen GHEJ Visser MGW Vosselman MJ . Cold acclimation recruits human brown fat and increases nonshivering thermogenesis. J Clin Invest. (2013) 123:3395–403. doi: 10.1172/JCI68993 23867626 PMC3726172

[41] Wang W Seale P . Control of brown and beige fat development. Nat Rev Mol Cell Biol. (2016) 17:691–702. doi: 10.1038/nrm.2016.96 27552974 PMC5627770

[42] Ikeda K Yamada T . UCP1 dependent and independent thermogenesis in brown and beige adipocytes. Front Endocrinol (Lausanne). (2020) 11:498. doi: 10.3389/fendo.2020.00498 32849287 PMC7399049

[43] Heyde I Begemann K Oster H . Contributions of white and brown adipose tissues to the circadian regulation of energy metabolism. Endocrinology. (2021) 162:bqab009. doi: 10.1210/endocr/bqab009 33453099 PMC7864004

[44] Sun Q Cui X Yin D Li J Li J Du L . Molecular mechanisms of UCP1-independent thermogenesis: the role of futile cycles in energy dissipation. J Physiol Biochem. (2025) 81:521–37. doi: 10.1007/s13105-025-01090-x 40380026

[45] Greenhill C . The role of brown adipose tissue in metabolic health. Nat Rev Endocrinol. (2024) 20:386. doi: 10.1038/s41574-024-00999-5 38745105

[46] Hachemi I U-Din M . Brown adipose tissue: activation and metabolism in humans. Endocrinol Metab. (2023) 38:214–22. doi: 10.3803/EnM.2023.1659 36972706 PMC10164504

[47] Gupta RK . Human brown fat and metabolic disease: a heated debate. J Clin Invest. (2023) 133:e176678. doi: 10.1172/JCI176678 38038128 PMC10688977

[48] Hropot T Herman R Janez A Lezaic L Jensterle M . Brown adipose tissue: a new potential target for glucagon-like peptide 1 receptor agonists in the treatment of obesity. Int J Mol Sci. (2023) 24:8592. doi: 10.3390/ijms24108592 37239935 PMC10218520

[49] Okamatsu-Ogura Y Kuroda M Tsutsumi R Tsubota A Saito M Kimura K . UCP1-dependent and UCP1-independent metabolic changes induced by acute cold exposure in brown adipose tissue of mice. (2020) 113:154396. doi: 10.1016/j.metabol.2020.154396 33065161

[50] Betz MJ Enerbäck S . Targeting thermogenesis in brown fat and muscle to treat obesity and metabolic disease. Nat Rev Endocrinol. (2018) 14:77–87. doi: 10.1038/nrendo.2017.132 29052591

[51] Negroiu CE Tudorașcu I Bezna CM Godeanu S Diaconu M Danoiu R . Beyond the cold: activating brown adipose tissue as an approach to combat obesity. J Clin Med. (2024) 13:1973. doi: 10.3390/jcm13071973 38610736 PMC11012454

[52] Bombassaro B Batitucci G Reymond Simoes M Araujo EP Velloso LA . The impact of dietary factors on the function of brown and beige adipose tissues-implications on health and disease. Front Nutr. (2025) 12:1626068. doi: 10.3389/fnut.2025.1626068 40832640 PMC12358295

[53] Singh R Barrios A Dirakvand G Pervin S . Human brown adipose tissue and metabolic health: potential or therapeutic avenues. Cell. (2021) 10:3030. doi: 10.3390/cells10113030 34831253 PMC8616549

[54] Wang F Qiao H Zhou H Zheng Y Ni Y He X . Thermogenic adipose tissue in older adults with obesity: a narrative review of mechanisms, brown fat resistance, and the translational relevance of exercise and nutrition. Front Nutr. (2026) 13:1818342. doi: 10.3389/fnut.2026.1818342 42221769 PMC13216019

[55] Itoh N Ohta H Konishi M . Endocrine FGFs: evolution, physiology, pathophysiology, and pharmacotherapy. Front Endocrinol (Lausanne). (2015) 6:154. doi: 10.3389/fendo.2015.00154 26483756 PMC4586497

[56] Kristóf E Klusóczki Á Veress R Shaw A Sándor Combi Z Varga K . Interleukin-6 released from differentiating human beige adipocytes improves browning. Exp Cell Res. (2019) 377:47–55. doi: 10.1016/j.yexcr.2019.02.015 30794803

[57] Chen Z Liu X Wang Y Zhang J Liu W . The secreted peptide BATSP1 promotes thermogenesis and improves glucose metabolism in adipocytes. Cell Mol Life Sci. (2023) 80:377. doi: 10.1007/s00018-023-05027-9 38010450 PMC10682272

[58] Yan C Burley G Gao H Shi Y-C . Brown adipose tissue crosstalk with pancreatic β-cells. Endocrinology. (2025) 166:bqaf118. doi: 10.1210/endocr/bqaf118 40658727 PMC12287633

[59] Ahmad B Vohra MS Saleemi MA Serpell CJ Fong IL Wong EH . Brown/Beige adipose tissues and the emerging role of their secretory factors in improving metabolic health: The batokines. Biochimie. (2021) 184:26–39. doi: 10.1016/j.biochi.2021.01.015 33548390

[60] Shamsi F Xue R Lian Huang T Lundh M Liu Y Leiria LO . FGF6 and FGF9 regulate UCP1 expression independent of brown adipogenesis. Nat Commun. (2020) 11:1421. doi: 10.1038/s41467-020-15055-9 32184391 PMC7078224

[61] Ng R Hussain NA Zhang Q Chang C Li H Fu Y . miRNA-32 drives brown fat thermogenesis and trans-activates subcutaneous white fat browning in mice. Cell Rep. (2017) 19:1229–46. doi: 10.1016/j.celrep.2017.04.035 28494871 PMC5637386

[62] Sponton CH Hosono T Taura J Jedrychowski MP Yoneshiro T Wang Q . The regulation of glucose and lipid homeostasis via PLTP as a mediator of BAT-liver communication. EMBO Rep. (2020) 21:e49828. doi: 10.15252/embr.201949828 32672883 PMC7507062

[63] BonDurant LD Ameka M Naber MC Jedrychowski MP Yoneshiro T Wang Q . FGF21 regulates metabolism through adipose-dependent and -independent mechanisms. Cell Metab. (2017) 25:935–944.e4. doi: 10.1016/j.cmet.2017.03.005 28380381 PMC5494834

[64] Ziqubu K Dludla PV Mabhida SE Jack BU Keipert S Jastroch M . Brown adipose tissue-derived metabolites and their role in regulating metabolism. Metabolism. (2024) 150:155709. doi: 10.1016/j.metabol.2023.155709 37866810

[65] Dong M Gao C Jia Y Xu W Liu Y Wen X . Temporal specificity of IL-6 knockout in enhancing the thermogenic capability of brown adipose tissue. J Physiol Biochem. (2022) 78:619–28. doi: 10.1007/s13105-021-00847-4 35349101

[66] Ding S Xu J-L Tong J-Y Cheng Y-Y Shi L-F Wei W . Brown adipose tissue alleviates podocyte apoptosis through NRG4 in a male mouse model of diabetic kidney disease. Diabetologia. (2025) 68:1057–75. doi: 10.1007/s00125-02506385-8 40014139

[67] Jena J García-Peña LM Weatherford ET Marti A Bjorkman SH Kato K . GDF15 is required for cold-induced thermogenesis and contributes to improved systemic metabolic health following loss of OPA1 in brown adipocytes. Elife. (2023) 12:e86452. doi: 10.7554/eLife.86452 37819027 PMC10567111

[68] Villarroya F . Brown adipose tissue in obesity and diabetes: pathophysiology and therapeutic potential. Diabetologia. (2018) 61:65–372.

[69] McNeill BT Suchacki KJ Stimson RH . Human brown adipose tissue as a therapeutic target: warming up or cooling down? Eur J Endocrinol. (2021) 184:R243–59. doi: 10.1530/EJE-20-1439 33729178 PMC8111330

[70] Alcalá M Calderon-Domínguez M Serra D Herrero L Viana M . Mechanisms of impaired brown adipose tissue recruitment in obesity. Front Physiol. (2019) 10:94. doi: 10.3389/fphys.2019.00094 30814954 PMC6381290

[71] Ishida Y Matsushita M Yoneshiro T Saito M Fuse S Hamaoka T . Genetic evidence for involvement of β2-adrenergic receptor in brown adipose tissue thermogenesis in humans. Int J Obes (Lond). (2024) 48:1110–7. doi: 10.1038/s41366-024-01522-6 38632325 PMC11281906

[72] Enerbäck S . Human brown adipose tissue. Cell Meta. (2010) 11:248–52. doi: 10.1016/j.cmet.2010.03.008 20374955

[73] Stanford KI Goodyear LJ . The therapeutic potential of brown adipose tissue. Hepatobiliary Surg Nutr. (2013) 2:286–7. doi: 10.3978/j.issn.2304-3881.2013.09.02 24570961 PMC3924699

[74] Ziqubu K Mazibuko-Mbeje SE Dludla PV . Regulation of adipokine and batokine secretion by dietary flavonoids, as a prospective therapeutic approach for obesity and its metabolic complications. Biochimie. (2025) 230:95–113. doi: 10.1016/j.biochi.2024.11.007 39551425

[75] Ruiz-Ojeda FJ Wang J Bäcker T Krueger M Zamani S Rosowski S . Active integrins regulate white adipose tissue insulin sensitivity and brown fat thermogenesis. Mol Metab. (2021) 45:101147. doi: 10.1016/j.molmet.2020.101147 33359386 PMC7808956

[76] Della Guardia L Shin AC . Obesity-induced tissue alterations resist weight loss: a mechanistic review. Diabetes Obes Metab. (2024) 26:3045–57. doi: 10.1111/dom.15637 38720199

[77] Cypess AM Weiner LS Roberts-Toler C Franquet Elía E Kessler SH Kahn PA . Activation of human brown adipose tissue by a β3-adrenergic receptor agonist. Cell Metab. (2015) 21:33–8. doi: 10.1016/j.cmet.2014.12.009 25565203 PMC4298351

[78] Sampath SC Sampath SC Bredella MA Cypess AM Torriani M . Imaging of brown adipose tissue: state of the art. Radiology. (2016) 280:4–19. doi: 10.1148/radiol.2016150390 27322970 PMC4942993

[79] Tanoue T Nagayama M A Roochana AJ Zimmerman S Ashenberg O Jain T . Microbiota-mediated induction of beige adipocytes in response to dietary cues. Nature. (2026) 653:499–509. doi: 10.1038/s41586-026-10205-3 41781619 PMC13051337

[80] Mirmiranpour H Barber TM . The role of batokines in metabolism and their therapeutic potential. Expert Rev Endocrinol Metab. (2026) 21:143–51. doi: 10.1080/17446651.2026.2624400 41631747

[81] Iwen KA Backhaus J Cassens M Waltl M Hedesan OC Merkel M . Cold-induced brown adipose tissue activity alters plasma fatty acids and improves glucose metabolism in men. J Clin Endocrinol Metab. (2017) 102:4226–34. doi: 10.1210/jc.2017-01250 28945846

[82] Duerre DJ Hansen JK John SV Jen A Carrillo ND Bui H . Haem biosynthesis regulates BCAA catabolism and thermogenesis in brown adipose tissue. Nat Metab. (2025) 7:1018–33. doi: 10.1038/s42255-025-01253-6 40133548 PMC12116240

[83] Echeverría F Bustamante A Sambra V Álvarez D Videla L Valenzuela R . Beneficial effects of dietary polyphenols in the prevention and treatment of NAFLD: cell-signaling pathways underlying health effects. Curr Med Chem. (2022) 29:299–328. doi: 10.2174/0929867328666210825111350 34525916

[84] Dakic T Velickovic K Lakic I Ruzicic A Milicevic A Plackic N . Rat brown adipose tissue thermogenic markers are modulated by estrous cycle phases and short-term fasting. Biofactors. (2024) 50:101–13. doi: 10.1002/biof.1993 37482913

[85] Mestres-Arenas A Quesada-López T Blasco-Roset A Giralt M Villarroya F Planavila A . Differential effects of the anti-obesity drug tirzepatide on adipose tissues: brown fat as a key target. Biomedicine Pharmacotherapy. (2026) 195:119057. doi: 10.1016/j.biopha.2026.119057 41592522

[86] Beiroa D Imbernon M Gallego R Senra A Herranz D Villarroya F . GLP-1 agonism stimulates brown adipose tissue thermogenesis and enhances energy expenditure. Diabetes. (2014) 63:4089–99. doi: 10.2337/db14-0302 24917578

[87] Blasco-Roset A Quesada-López T Mestres-Arenas A Villarroya J Godoy-Nieto FJ Cereijo R . Acyl CoA binding protein controls brown adipose tissue thermogenesis and systemic metabolism. Mol Metab. (2025) 89:102153. doi: 10.1016/j.molmet.2025.102153 40220929 PMC12050000

[88] Li Y . CLCF1 is a brown adipose tissue derived cytokine that suppresses thermogenesis and impairs metabolic homeostasis’. Proc Natl Acad Sci. (2024) 121:e2310711121. doi: 10.1073/pnas.2310711121 38190531 PMC10801846

[89] Rui L . Brown and beige adipose tissues in health and disease. Compr Physiol. (2017) 7:1281–306. doi: 10.1002/cphy.c170001 28915325 PMC6192523

[90] Maliszewska K Kretowsk A . Brown adipose tissue and its role in insulin and glucose homeostasis. Int J Mol Sci. (2021) 22:1530. doi: 10.3390/ijms22041530 33546400 PMC7913527

[91] Chondronikola M Volpi E Børsheim E Porter C Annamalai P Enerbäck S . Brown adipose tissue improves whole-body glucose homeostasis and insulin sensitivity in humans. Diabetes. (2014) 63:4089–99. doi: 10.2337/db14-0746 25056438 PMC4238005

[92] Ravussin E Galgani JE . The implication of brown adipose tissue for humans. Annu Rev Nutr. (2011) 31:33–47. doi: 10.1146/annurev-nutr-072610-145209 21548774 PMC4404503

[93] Xiong G Zhang K Ma Y . BAM15 as a mitochondrial uncoupler: a promising therapeutic agent for diverse diseases. Front Endocrinol. (2023) 14:1252141. doi: 10.3389/fendo.2023.1252141 37900126 PMC10600450

[94] Souza Magro B Penteado Martins Dias D . Brown and beige adipose tissue: new therapeutic targets for metabolic disorders. Health Sci Rev. (2024) 10:100148. doi: 10.1016/j.hsr.2024.10014842574925

[95] Yoshida Y Shimizu I Shimada A Nakahara K Yanagisawa S Kubo M . Brown adipose tissue dysfunction promotes heart failure via a trimethylamine N-oxide-dependent mechanism. Sci Rep. (2022) 12:14883. doi: 10.1038/s41598-022-19245-x 36050466 PMC9436957

[96] Becher T Palanisamy S Kramer DJ Eljalby M Marx SJ Wibmer AG . Brown adipose tissue is associated with cardiometabolic health. Nat Med. (2021) 27:58–65. doi: 10.1038/s41591-020-1126-7 33398160 PMC8461455

[97] Challa AA Vidal P Maurya SK Maurya CK Baer LA Wang Y . UCP1-dependent brown adipose activation accelerates cardiac metabolic remodeling and reduces initial hypertrophic and fibrotic responses to pathological stress. FASEB J. (2024) 38:e23709. doi: 10.1096/fj.202400922R 38809700 PMC11163965

[98] Zhao Y Chen H Zhang T . Brown adipose tissue-derived FGF21 mediates the cardioprotection of dexmedetomidine in myocardial ischemia/reperfusion injury. Sci Rep. (2024) 14:18292. doi: 10.1038/s41598-024-69356-w 39112671 PMC11306229

[99] Pinckard KM Shettigar VK Wright KR Abay E Baer LA Vidal P . A novel endocrine role for the BAT-released lipokine 12,13-diHOME to mediate cardiac function. Circulatio. (2021) 143:145–59. doi: 10.1161/CIRCULATIONAHA.120.049813 33106031 PMC7856257

[100] Pinckard KM Stanford KI . The heartwarming effect of brown adipose tissue. Mol Pharmacol. (2022) 102:460–71. doi: 10.1124/molpharm.121.000328 34933905 PMC9341250

[101] Ying Z Tramper N Zhou E Boon MR Rensen PCN Kooijman S . Role of thermogenic adipose tissue in lipid metabolism and atherosclerotic cardiovascular disease: Lessons from studies in mice and humans. Cardiovasc Res. (2023) 119:905–18. doi: 10.1093/cvr/cvac131 35944189 PMC10153643

[102] Thoonen R Ernande L Cheng J Nagasaka Y Yao V Miranda-Bezerra A . Functional brown adipose tissue limits cardiomyocyte injury and adverse remodeling in catecholamine-induced cardiomyopathy. J Mol Cell Cardiol. (2015) 84:202–11. doi: 10.1016/j.yjmcc.2015.05.002 25968336 PMC4470477

[103] Dixon AE Peters UTP . Adipose tissue inflammation and pulmonary dysfunction in obesity. Int J Mol Sci. (2022) 23:7349. doi: 10.3390/ijms23137349 35806353 PMC9267094

[104] Papaioannou O Karampitsakos T Barbayianni I Chrysikos S Xylourgidis N Tzilas V . Metabolic disorders in chronic lung diseases. Front Med (Lausanne). (2018) 4:246. doi: 10.3389/fmed.2017.00246 29404325 PMC5778140

[105] Suratt BT Ubags NDJ Rastogi D Tantisira KG Marsland BJ Petrache I . An official American Thoracic Society workshop report: Obesity and metabolism. An emerging frontier in lung health and disease. Ann Am Thorac Soc. (2017) 14:1050–9. doi: 10.1513/AnnalsATS.201703-263WS 28570148 PMC5802569

[106] Wright TC Stanford KI . Batokines: Mediators of inter-tissue communication (a mini-review). Curr Obes Rep. (2022) 11:1–9. doi: 10.1007/s13679-021-00465-7 34997461 PMC9165237

[107] Kerstholt N Ewert R Nauck M Spielhagen T Bollmann T Stubbe B . Association of circulating irisin and cardiopulmonary exercise capacity in healthy volunteers: Results of the Study of Health in Pomerania. BMC Pulm Med. (2015) 15:41. doi: 10.1186/s12890-015-0035-x 25898830 PMC4416285

[108] Castillo-Armengol J Fajas L Lopez-Mejia IC . Inter-organ communication: A gatekeeper for metabolic health. EMBO Re. (2019) 20:e47903. doi: 10.15252/embr.201947903 31423716 PMC6726901

[109] Miricescu D Balan DG Tulin A Stiru O Vacaroiu IA Mihai DA . Impact of adipose tissue in chronic kidney disease development (review). (2021) 21:539. doi: 10.3892/etm.2021.9969 PMC8014972 33815612

[110] Cai Y-Y Zhang H-B Fan C-X Zeng Y-M Zou S-Z Wu C-Y . Renoprotective effects of brown adipose tissue activation in diabetic mice. J Diabetes. (2019) 11:958–70. doi: 10.1111/1753-0407.12938 31020790 PMC6899899

[111] Feng L Cui J Chen W Zhu L Li P Zhou H . Neuregulin 4 secreted by brown adipose tissue suppresses ferroptosis of sepsis-induced liver injury. Inflammation. (2025) 48:2783–801. doi: 10.1007/s10753-024-02230-z 39956880 PMC12336088

[112] Chondronikola M Volpi E Børsheim E Porter C Saraf MK Annamalai P . Brown adipose tissue activation is linked to distinct systemic effects on lipid metabolism in humans. Cell Metab. (2016) 23:1200–6. doi: 10.1016/j.cmet.2016.04.029 27238638 PMC4967557

[113] Fan M Wang Y Jin L Fang Z Peng J Tu J . Bile acid-mediated activation of brown fat protects from alcohol-induced steatosis and liver injury in mice. Cell Mol Gastroenterol Hepatol. (2022) 13:809–26. doi: 10.1016/j.jcmgh.2021.12.001 34896286 PMC8802063

[114] Worthmann A John C Rühlemann MC . Cold-induced conversion of cholesterol to bile acids in mice shapes the gut microbiome and promotes adaptive thermogenesis. Nat Med. (2017) 23:839–49. doi: 10.1038/nm.4357 28604703

[115] Zangerolamo L Carvalho M Barbosa HCL . The critical role of the bile acid receptor TGR5 in energy homeostasis: Insights into physiology and therapeutic potential. Int J Mol Sci. (2025) 26:6547. doi: 10.3390/ijms26146547 40724796 PMC12294878

[116] Broeders EPM Nascimento EBM Havekes B Brans B Roumans KHM Tailleux A . The bile acid chenodeoxycholic acid increases human brown adipose tissue activity. Cell Metab. (2015) 22:418–26. doi: 10.1016/j.cmet.2015.07.002 26235421

[117] Chen X Lou G Meng Z Huang W . TGR5: A novel target for weight maintenance and glucose homeostasis. (2011) 2011:853501. doi: 10.1155/2011/853501 PMC3132465 21754919

[118] Yang J de Vries HD Mayeuf-Louchart A . Role of bile acid receptor FXR in development and function of brown adipose tissue. Biochim Biophys Acta Mol Cell Biol Lipids. (2023) 1868:159257. doi: 10.1016/j.bbalip.2022.159257 36402299

[119] Gavaldà-Navarro A Villarroya J Cereijo R Giralt M Villarroya F . The endocrine role of brown adipose tissue: An update on actors and actions. Rev Endocr Metab Disord. (2022) 23:31–41. doi: 10.1007/s11154-021-09640-6 33712997

[120] Ahmed BA Ong FJ Barra NG Blondin DP Gunn E Oreskovich SM . Lower brown adipose tissue activity is associated with non-alcoholic fatty liver disease but not changes in the gut microbiota. Cell Rep Med. (2021) 2:100397. doi: 10.1016/j.xcrm.2021.100397 34622234 PMC8484690

[121] Schlein C Heeren J . Implications of thermogenic adipose tissues for metabolic health. Best Pract Res Clin Endocrinol Metab. (2016) 30:487–96. doi: 10.1016/j.beem.2016.08.002 27697210

[122] Chouchani ET Kazak L Spiegelman BM . Mitochondrial reactive oxygen species and adipose tissue thermogenesis: Bridging physiology and mechanisms. J Biol Chem. (2017) 292:16810–6. doi: 10.1074/jbc.R117.789628 28842500 PMC5641863

[123] Leitner BP Weiner LS Desir M Kahn PA Selen DJ Tsang C . Kinetics of human brown adipose tissue activation and deactivation. Int J Obes (Lond). (2019) 43:633–7. doi: 10.1038/s41366-018-0104-3 29795459 PMC6252171

[124] Cannon B Nedergaard J . Brown adipose tissue: Function and physiological significance. Physiol Rev. (2004) 84:277–359. doi: 10.1152/physrev.00015.2003 14715917

[125] Tabuchi C Sul HS . Signaling pathways regulating thermogenesis. Front Endocrinol (Lausanne). (2021) 12:595020. doi: 10.3389/fendo.2021.595020 33841324 PMC8034539

[126] Cypess AM Haft CR Laughlin MR Hu HH . Brown fat in humans: Consensus points and experimental guidelines. Cell Metab. (2014) 20:408–15. doi: 10.1016/j.cmet.2014.07.025 25185947 PMC4155326

[127] Kwok TC Ramage LE Kelman A Suchacki KJ Gray C Boyle LD . UCP1 expression in human brown adipose tissue is inversely associated with cardiometabolic risk factors. Eur J Endocrinol. (2024) 191:106–15. doi: 10.1093/ejendo/lvae074 38917410 PMC11265601

[128] Kononova YA Tuchina TP Babenko AY . Brown and beige adipose tissue: One or different targets for treatment of obesity and obesity-related metabolic disorders? Int J Mol Sci. (2024) 25:13295. doi: 10.3390/ijms252413295 39769065 PMC11677471

[129] Karanfil AS Louis F Matsusaki M . Brown adipose tissue engineering: Advances, challenges, and future directions. Trends Biotechnol. (2026) 44:957–74. doi: 10.1016/j.tibtech.2025.08.010 41006179

[130] Hu HH Chen J Shen W . Segmentation and quantification of adipose tissue by magnetic resonance imaging. MAGMA. (2016) 29:259–76. doi: 10.1007/s10334-015-0498-z 26336839 PMC5206913

[131] Yan M Wang H Sha W Fu Y . UCP1-dependent thermogenic adipose tissue in human disease: Adipose-centered mechanisms, biomarker limitations, and translational perspectives. Int J Mol Sci. (2026) 27:6416. doi: 10.3390/ijms27146416 42511758 PMC13410023

[132] Zhao Z-W Liu Q Hu L Song B Wu Q Lin J . DNA-mediated UCP1 overexpression in adipose tissue: A promising anti-obesity gene therapy. Clin Transl Med. (2025) 15:e70491. doi: 10.1002/ctm2.70491 41039748 PMC12491148

